# Ileo-anal pouch necrosis secondary to small bowel volvulus: A case report

**DOI:** 10.1186/1749-7922-3-18

**Published:** 2008-05-30

**Authors:** Sandeep Patel, Gurcharan Salotera, Shashank Gurjar, Jim Hewes, Ibrahim Ahmed, Brian Andrews

**Affiliations:** 1 Department of Surgery, Medway Maritime Hospital, Gillingham, Kent, UK

## Abstract

**Introduction:**

Small bowel volvulus is a rare occurrence in the Western world and its occurrence after ileo-anal ouch formation is even rarer.

**Case Presentation:**

We report a case of a 26 year old lady who presented with small bowel volvulus and subsequent ischaemia and necrosis of her ileo-anal pouch created 5 years previously.

**Conclusion:**

This case illustrates a rare but potentially devastating complication of ileo-anal pouch formation and as such the diagnosis should be borne in mind when a patient with a pouch presents with an acute abdomen.

## Introduction

Small bowel volvulus (SBV) as a cause of intestinal obstruction is an uncommon surgical emergency. It is a recognised entity in neonates associated with abnormal midgut rotation[1,2], but is seldom seen in adults. Delayed diagnosis may lead to bowel ischaemia, infarction and consequent morbidity. We describe a case of acute small bowel volvulus and resultant pouch necrosis in a patient who had previously undergone total colectomy with ileo-anal pouch formation.

## Case Report

**Figure 1:**
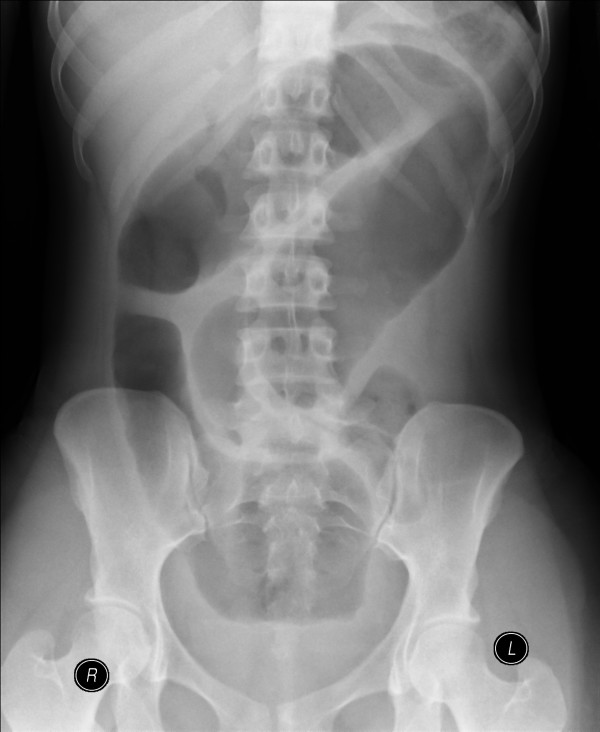
A loop of dilated small bowel mid abdomen with no clear valvulae coniventes continuing into the pelvis.

**Figure 2:**
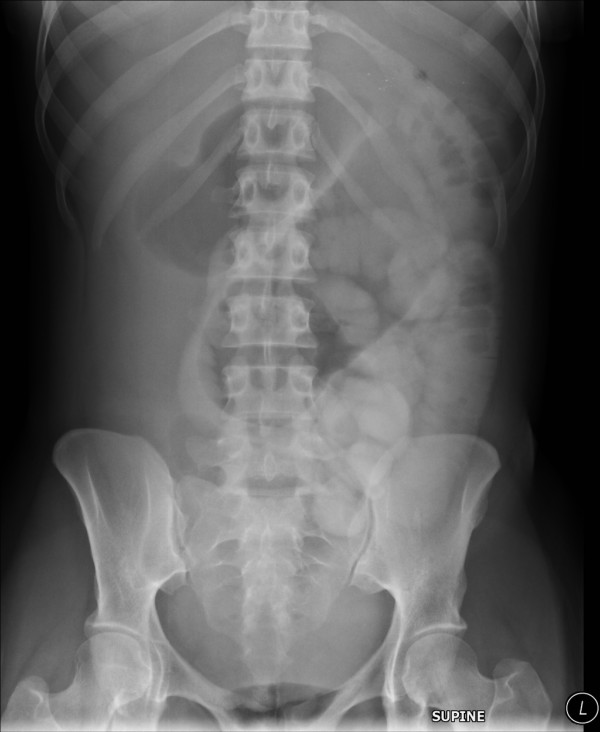
A six film hour post therapeutic gastrograffin consistent with mechanical small bowel obstruction.

**Figure 3:**
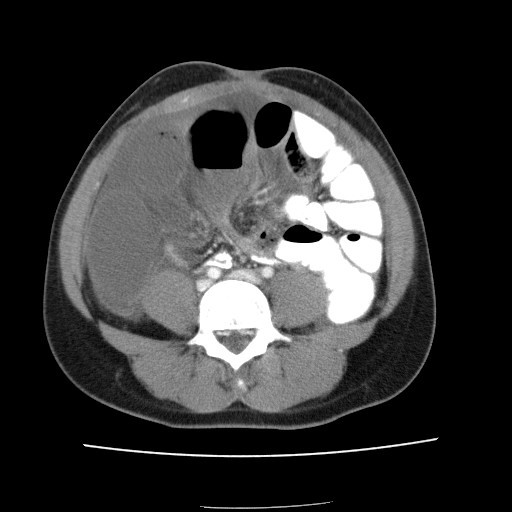
CT showing dilated proximal jejunum and a closed loop obstruction in the terminal ileum. There is a positive "swirl" sign consistent with a diagnosis of small bowel volvulus.

**Figure 4:**
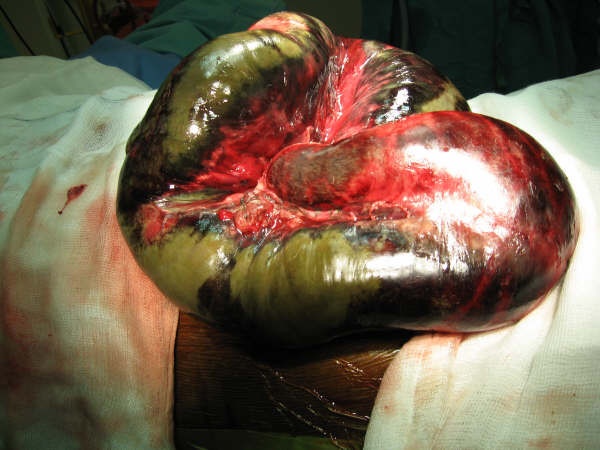
Intraoperative picture showing small bowel volvulus and necrotic bowel.

**Figure 5:**
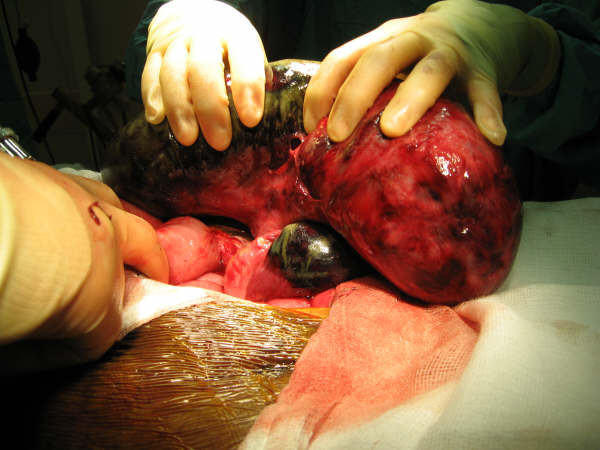
Intraoperative picture showing necrosis of ileo-anal pouch.

A 26-year old Afro-Caribbean lady presented acutely with a 24-hour history of colicky abdominal pain, fever and vomiting. She was known to have sickle cell trait and a history of ulcerative colitis, for which she had undergone a total colectomy with ileo-anal pouch formation 5 years earlier. On examination she had a distended abdomen, generalised abdominal tenderness and features suggestive of small bowel obstruction. The clinical diagnosis was confirmed on plain and contrast radiography (Figure 1, 2). An abdominal CT scan demonstrated a dilated proximal jejunum with closed loop obstruction of the terminal ileum and a positive 'corkscrew' sign (Figure 3). Therapeutic oral gastrografin was tried but failed. The patient deteriorated and proceeded to laparotomy. At surgery a section of small bowel proximal to the pouch was found to have twisted on its mesentery. This small bowel volvulus had caused a closed loop obstruction with consequent necrosis of both the section of twisted jejunum and the ileo-anal pouch (Figure 4, 5). The pouch was taken down and the sections of gangrenous bowel excised, and an end ileostomy fashioned. Histological analysis of the excised specimens confirmed the operative findings of small bowel gangrene. The patient made a slow post-operative recovery as a consequence of malabsorption secondary to short bowel syndrome.

## Discussion

SBV is a rare cause of small bowel obstruction in the West with a reported incidence of 1.7–5.7 cases per 100,000 habitants[1] and an associated mortality rate of upto 35%[3]. Primary SBV occurs when the small bowel rotates around its mesentery (in the absence of congenital or adhesive bands) causing interruption to the blood supply to and from the bowel resulting in venous congestion, obstruction, ischaemia and eventual necrosis. Various aetiological factors have been proposed in its development as a primary condition including diet and changes in gut motility[4]. Observational studies have noted an increased incidence of the condition during periods of feasting in the Third World where after starvation large amounts of food are rapidly ingested[5]. The displacement of the jejunum into the pelvis by a large food bolus may cause the empty small bowel to move proximally within the abdomen and cause volvulus. This requires the presence of a short and broad mesentery and firm abdominal wall muscles to prevent coronal movement of the bowel[6].

Changes in gut motility and tone have also been implicated in the formation of primary volvulus. Chaussade *et al *reported the presence of increased discrete clustered contractions after small bowel anastomosis to the anal sphincter, possibly as a consequence of functional obstruction[7]. Alterations in the levels of the gut motility stimulant 5-hydroxy-tryptamine (5-HT), as well as diabetic autonomic neuropathy have also been implicated[8,9].

Secondary SBV is more commonly seen in the West and can develop as a result of twisting of the small bowel around a fixed point that may have arisen due to adhesions[10], internal herniation [11], or Meckel's diverticulum[12]. An association between SBV and large small bowel diverticula (>3 cm) has also been noted [13] as well as an isolated report of SBV secondary to a migrating mesh plug[14]. The alteration of the relationship of the superior mesenteric artery to its corresponding vein, as a result of torsion of the mesentery around its attachment, and the associated 'swirl sign' are important markers of SBV[15].

Ileo-anal pouch formation is a commonly performed procedure for patients with ulcerative colitis to maintain faecal continence following colectomy. Complications of the procedure include the development of cuff abscess, pouch leak, strictures, fistulation, stenosis, and pouchitis[16]. Small bowel obstruction as a complication of ileo-anal pouch formation has been described in the literature, and is usually a result of post-operative adhesions either within the pelvis, or at the site of the covering ileostomy[17-20]. The development of pouch ischaemia and necrosis following small bowel volvulus however is a rare complication with only one reported case identified[10] with classical radiological 'corkscrew' findings as apparent in our case. This diagnosis should therefore be considered in patients presenting with small bowel obstruction following ileo-anal pouch procedures.

## Consent

The patient concerned has given her full informed consent for the use of all radiology and pictures and the case history in the production of this paper

## Competing interests

The authors declare that they have no competing interests.

## Authors' contributions

SP drafted and edited the manuscript

GS drafted part of the manuscript

SG edited the manuscript

JH edited the manuscript

IA performed the surgery

BA performed the surgery
